# Hypothesis-free evaluation of circulating metabolome provides cell-specific insights regarding the role of energy substrate availability in amyotrophic lateral sclerosis

**DOI:** 10.1186/s12916-026-04727-w

**Published:** 2026-03-06

**Authors:** Elham Alhathli, Johnathan Cooper-Knock, Zain-Ul-Abideen Girach, Thomas H. Julian, Claudia Bauer, Hannah O. Timmons, Billie D. Ward, Heather Walker, Mimoun Azzouz, Mohamed A. Elrayess, Fatima Al-Khelaifi, Noha A. Yousri, Aytac Gul, Alan Kelsall, Tobias Moll, Calum Harvey, Sarah Gornall, Kari Wong, Scott P. Allen, Andrew Strange, Pamela J. Shaw

**Affiliations:** 1 Sheffield Institute for Translational Neuroscience, University of Sheffield, Glossop Road, Sheffield, S10 2HQ UK; 2 Faculty of Nursing, Taif University, Taif, Saudi Arabia; 3 Division of Evolution, Infection and Genomics, School of Biological Sciences, The University of Manchester, Manchester, UK; 4 Christabel Pankhurst Institute, The University of Manchester, Manchester, UK; 5 Biomedical Research Center (BRC), QU Health, Qatar University, P.O. Box 2713, Doha, Qatar; 6 Anti-Doping Laboratory Qatar, Doha, Qatar; 7 Computer and Systems Engineering, Faculty of Engineering, Alexandria University, Alexandria, Egypt; 8 Department of Medical Biology, Tayfur Ata Sökmen Medical Faculty, Hatay Mustafa Kemal University, Antakya, Turkey; 9 School of Medicine and Population Health, University of Sheffield, Sheffield, UK; 10 Metabolon Inc, Durham, NC USA; 11 NIHR Sheffield Biomedical Research Centre (BRC), Sheffield Teaching Hospitals NHS Foundation Trust, Glossop Road, Sheffield, S10 2JF UK

**Keywords:** Amyotrophic lateral sclerosis, Metabolomics, Mendelian randomisation, Acetylcarnitine, Carnitine shuttle, Cerebrospinal fluid, Energy metabolism, Neuronal vulnerability, GLUT3

## Abstract

**Background:**

Amyotrophic lateral sclerosis (ALS) is a neurodegenerative disease with limited therapeutic options. The circulating metabolome comprises small molecules present in plasma/serum which are the intermediates and end-products of cellular metabolism, and is linked to ALS pathogenesis.

**Methods:**

We conducted hypothesis-free two-sample Mendelian randomisation (MR) analysis of the concentration of 575 plasma/serum metabolites, to determine which are causally linked to risk of ALS. Significant metabolites were validated in an independent GWAS of plasma/serum metabolite concentrations and evaluated for sex-specific effects. Correlations between directly measured patient biofluid metabolite concentrations and ALS risk/severity were examined in 94 ALS patients and 40 controls. We experimentally assessed metabolic function in a murine neurons and human astrocytes carrying an ALS-associated G4C2-repeat expansion within *C9orf72*.

**Results:**

MR causally associated five metabolites with ALS risk after multiple-testing correction. Higher serum concentration of glycoprotein acetyls (*P* = 9.7e − 9, *β* = 0.21) and the peptide DSGEGDFXAEGGGVR (*P* = 8.0e − 6, *β* = 0.22) was associated with increased ALS risk, whereas higher plasma concentration of phenylalanylserine, isobutyrylcarnitine, and acetylcarnitine was protective (*P* < 5e − 5, *β* = − 0.29 to − 0.72). DSGEGDFXAEGGGVR has been linked to glucose metabolism but we have used genetic fine-mapping to link DSGEGDFXAEGGGVR, neuronal glucose uptake through GLUT3, and ALS risk. Direct measurement of metabolite concentrations in patient biofluids revealed elevated acetylcarnitine levels in patients with ALS, which were associated with delayed symptom onset (Cox regression, *P* = 0.02, *HR* = 0.4). Similarly, lactate is elevated in ALS patient CSF (ANOVA, *P* = 1.3e − 3) and in patients with longer survival time (Cox regression, *P* = 0.03, *HR* = 0.3). Plasma fructose is elevated in ALS patients with shorter survival time (Cox regression, *P* = 0.02, *HR* = 1.1). In vitro, neurons and astrocytes carrying an ALS-associated G4C2-repeat expansion within *C9orf72* demonstrated reduced metabolic flexibility.

**Conclusions:**

We provide evidence that impaired energy substrate availability contributes to ALS risk and severity. CNS cell types differ in their use of energy substrates and therefore we postulate the relative importance of different cell types for different stages of disease. Our findings support further investigation of metabolic interventions to treat or prevent ALS.

**Supplementary Information:**

The online version contains supplementary material available at 10.1186/s12916-026-04727-w.

## Background

Amyotrophic lateral sclerosis (ALS), a rapidly progressive and invariably fatal neurodegenerative disorder, is characterised by the progressive loss of motor neurons (MN) [1]. The majority of ALS is thought to result from complex gene–environment interactions. Discovery of these gene–environment interactions could lead to personalised medicine approaches.

The metabolome is the complete set of small-molecule metabolites (typically < 1 kDa) present in biological tissues or fluids, reflecting the end-products and intermediates of cellular metabolism. This includes endogenous or exogenous molecules such as amino acids, nucleic acids, fatty acids, and environmental substrates [2]. As such, the metabolome is subject to both genetic and environmental influences; for example, the concentration of a particular metabolite might be influenced by both genetically determined enzyme activity and the availability of environmental substrate. The metabolome has been linked to both ALS risk and severity [3, 4].

Mendelian randomisation (MR) is a method whereby genetic data are used to perform causal inference between exposures, such as serum metabolite concentration, and outcomes, such as ALS risk. MR results are contingent on a set of three assumptions. A strong correlation must exist between the exposure and instrumental single-nucleotide polymorphisms (SNPs). SNPs should influence disease risk solely via the exposure, and SNPs must be uncorrelated with potential confounders which are related to both the exposure and the outcome [5]. MR relies on genetic changes that are fixed at conception; such changes are certainly upstream of ALS, which is a late age at onset disease. Significantly, two-sample MR does not require the exposure and outcome to be measured in the same sample set, which facilitates the use of large datasets with significant statistical power [5]. This makes MR an attractive approach for causal inference in ALS, although it can be difficult to exclude the possibility of confounding factors correlated with both the tested exposure and ALS risk.

**Fig. 1:**
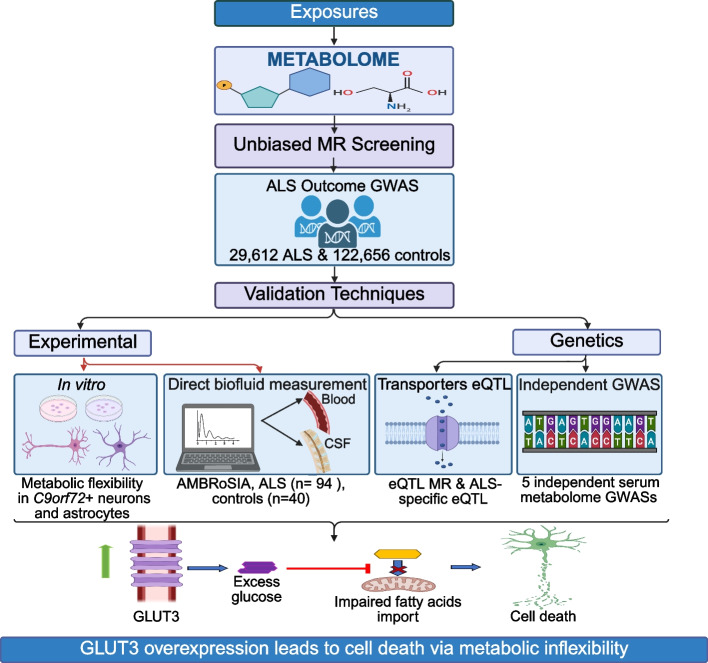
Multilayered analysis provides convergent evidence that impaired energy substrate availability contributes to ALS risk and severity. Hypothesis-free MR analysis causally associated the circulating concentration of multiple energy substrates with ALS risk, a result confirmed by MR using independent GWAS datasets and via direct measurement of metabolite concentrations in plasma and CSF. In vitro, neurons and astrocytes carrying the *C9orf72* G4C2-repeat expansion, a risk factor for ALS, are impaired in their ability to utilise diverse energy substrates. Supporting the idea that this inflexibility is disease-relevant, genetic determinants of neuronal glucose uptake ('transporters eQTL') modify risk for ALS

In a previous study, we conducted a hypothesis-free MR study of the circulating metabolome to determine which metabolite concentrations are causally associated with risk of ALS [6]. In the current study, we extended this approach using modified methodology and a new, larger ALS genome-wide association study (GWAS) comprising 29,612 ALS cases and 122,656 controls [7]. To mitigate the possibility that our MR findings are affected by an unseen confounder, we leveraged several orthogonal analyses to support our MR findings. We validated causally associated metabolites through direct measurement of plasma and cerebrospinal fluid (CSF) metabolite concentrations in 94 patients with ALS and 40 controls. Overall, we concluded that the availability of energy substrates used by disease-relevant CNS cell types is causally linked to ALS risk and severity. Astrocytes, which rely on fatty acid β-oxidation, appear to be particularly important determinants of disease onset whereas neurons and microglia may be determinants of survival time. We hypothesised that energy substrate availability interacts synergistically with a selective vulnerability of patient cells to energy deficit. Consistent with this, we experimentally demonstrated that cortical neurons and astrocytes carrying the ALS-associated *C9orf72* G4C2-repeat expansion [8] are impaired in their ability utilise diverse energy substrates. Finally, we fine-mapped SNPs linked to glucose metabolism and discovered that ALS risk is specifically linked to glucose uptake via the neuron-specific GLUT3 transporter protein, making this an attractive therapeutic target for ALS. Our approach is summarised in Fig. 1.

## Methods

### Exposure and outcome GWAS

We used two-sample MR to determine whether metabolite exposures are causally linked to ALS risk in the largest available ALS GWAS with 29,612 cases and 122,656 controls [7].

We performed an analysis of the effect of 575 circulating metabolites on ALS risk using two publicly available GWASs of the serum/plasma metabolome [9, 10]; we designated this analysis to be a ‘MR screen’. Both studies involved an almost exclusively European population and included approximately 2000 individuals from the KORA cohort (Cooperative Health Research in the Region of Augsburg). However, they differed in sample size and methodology: Kettunen et al. employed nuclear magnetic resonance (NMR) spectroscopy to quantify 123 metabolites in serum samples from up to 24,925 individuals [10], whereas Shin et al. employed liquid-phase and gas chromatography coupled with tandem mass spectroscopy to quantify 452 metabolites in plasma/serum from 7824 individuals [9]. The methodologies were complementary: NMR allows high-throughput profiling but has lower sensitivity than mass spectroscopy-based quantification [11]. Sixteen (4%) metabolites overlapped across the two studies; we did not remove any measures from the final set of tests but none of these 16 metabolites was among our five top candidates.

### Genetic instruments and MR framework

Genetic instruments used in the MR screen were selected based on a *P* value of 5e − 8 to 5e − 6; the lowest threshold was chosen to ensure more than five instrumental SNPs. When the cutoff is too low, informative instruments will be lost, but when it is too high, noninformative instruments will be introduced, and instrument pleiotropy is more likely to occur [6]. Only metabolites with ≥ 5 LD-independent SNPs were included in MR analyses. Independent SNPs were clumped using a stringent cutoff of *R* < 0.001 within a 10,000-kb window in the European reference panel. For clumped SNPs in linkage disequilibrium (LD), the SNP with the lowest *P* value was retained. When an exposure SNP was unavailable in the outcome dataset, a proxy with a high degree of LD (*R*^*2*^ > 0.9) within a European reference population was identified; if no suitable proxy was available, the SNP was excluded from the instrument set. SNP effects on outcomes and exposures were harmonised so that the *β* values were based on the same alleles. To reduce the risk of errors due to strand issues, palindromic alleles with minor allele frequency (MAF) > 0.42 were excluded from the analysis [12].

For each metabolite studied in our MR screen, we performed an MR using a multiplicative random-effects inverse-variance weighted (IVW) estimate for significance testing, as this method has the greatest statistical power [13] and enables accurate causal inferences for ≥ 5 instrumental SNPs under the assumption of limited balanced pleiotropy. To control the family-wise error rate for the hypothesis-free metabolome-wide MR screen, we applied a Bonferroni correction, dividing *α* = 0.05 by the total number of metabolites tested (*n* = 575), resulting in a significance threshold of *P* < 8.7 × 10^−5^ to identify metabolites causally associated with ALS.

Metabolites causally associated with ALS risk in our MR screen were validated by additional MR using different independent exposure GWASs to derive genetic instruments. Outcome GWAS and instrument-selection criteria remained constant. For acetylcarnitine and isobutyrylcarnitine, validation GWAS included 7478 individuals of European ancestry [14], a GWAS of 490 Qatari and Italian athletes [15], and a GWAS of 8262 Canadians of European descent [16]. The validation GWAS for DSGEGDFXAEGGGVR included 14,296 people of European ancestry [17].

For acetylcarnitine, we also employed a sex-specific analysis. This required a sex-specific ALS outcome GWAS reported previously [18]. The male-specific GWAS included 15,547 ALS cases and 50,145 controls, and the female-specific GWAS included 10,895 ALS cases and 57,062 controls.

### Robustness and sensitivity analyses

To increase confidence in the IVW results from our unbiased MR screen, we performed several robust MR measures and sensitivity analyses. We used an *F*-statistic to measure the strength of the association between instrumental SNPs and the exposure of interest. An *F*-statistic > 10 indicates that a SNP-derived estimate has a bias of < 10% regarding its intragroup variability and signifies an acceptable instrument. Pleiotropy occurs between SNPs when the difference in effect size for the exposure is not proportional to the difference in effect size for the outcome; it usually results from a violation of one of the key assumptions underlying MR (namely, that instrumental SNPs should be associated with the outcome only through the exposure) [5]. To account for pleiotropy, we removed SNPs when the *P* value for the association with the outcome was lower than for the association with the exposure of interest. The MR-Egger intercept test was also used to identify directional horizontal pleiotropy. To avoid false positives, we limited our analysis to exposures with ≥ 5 instrumental SNPs and used a q-q plot to determine whether the median *P* value was significantly inflated. A test with too few SNPs gives excessive weight to each individual SNP [19]. Because IVW estimates are vulnerable to pleiotropic SNPs, we used Cochran’s *Q* test (*P* > 0.05) along with the multiplicative random-effects IVW as a sensitivity measure to detect heterogeneity indicating pleiotropy. Moreover, radial MR [20] was used to remove statistically significant outlier SNPs. The *I*^2^ statistic was used to measure the heterogeneity between variant-specific causal estimates, with a low *I*^2^ indicating bias towards the null hypothesis [21]. A leave-one-out (LOO) analysis was applied to identify results when one or more SNPs exerted a disproportionate effect. TwoSampleMR (version 0.5.6), Mendelian Randomization (version 0.5.1), and RadialMR (version 1.0) R packages were used for all MR analyses.

### Genetic study of plasma DSGEGDFXAEGGGVR concentration

The variant rs10846162 is associated with circulating concentration of DSGEGDFXAEGGGVR, GLUT3 expression in motor neurons, and with ALS risk. Phenotypic associations were examined in the Project MinE whole-genome sequencing cohort (datafreeze 2: 6538 ALS cases and 2415 controls) [7] using logistic regression with age, sex, and the first ten principal components of genetic variation as covariates; site-of-onset analyses used the same model restricted to cases. All tests were two-sided, with statistical significance defined as *P* < 0.05.

### Validation via direct measurement of plasma metabolites in patients with ALS and controls

Blood plasma and cerebrospinal fluid (CSF) samples were obtained from 94 patients and 40 controls, as part of the AMBRoSIA project. A total of 1659 metabolites were surveyed in plasma, whereas 517 were assessed in CSF using ultra-high-performance liquid chromatography-tandem mass spectrometry (Metabolon, Inc); using both reverse-phase and hydrophilic interaction chromatography in both positive and negative ion modes. Recovery and internal standards were included in the plasma samples to assess extraction efficiency and instrument performance. Instrument precision was monitored with a pooled technical replicate, which was injected periodically to gauge platform variability. Metabolites were identified based on mass-to-charge ratio, retention time, and chromatographic properties matched against a library of validated standards. Daily variability was corrected by normalising metabolite intensities to the median for each day, with values scaled accordingly within each sample. For missing values, the lowest concentration observed for each metabolite across the cohort was substituted. Readings were normalised via batch normalisation: raw measurement values were divided by the median of all samples in each sample batch, giving each batch and thus the metabolite a median of 1. Sample donor information can be found in Supplementary Table 7 for both controls and ALS donors.

Data analysis was performed using R. Briefly, Cox proportional hazard analysis and Kaplan–Meier curves were applied using ‘coxph’ and ‘surv’ functions from the ‘survival’ package (V3.7–0) [22]. Unless otherwise noted, multiple-testing correction was performed using the Benjamini–Hochberg procedure to control the false discovery rate (FDR). This is distinct from the primary hypothesis-free MR screen where we applied a strict Bonferroni correction recognising that this step included a larger number of statistical tests and a lower pre-test probability.

### Cortical neuron culture

Cortical neurons were isolated from six E18 embryonic C9orf72-BAC mice (C57BL/6) [8] and six nontransgenic control mice; both groups were sex balanced. Neurons were cultured on poly-D-ornithine/poly-L-lysine half-area 96-well plates (Greiner) or 24-well Seahorse plates (Agilent) at 25,000 cells per well or 40,000 cells per well, respectively, in NeurobasalA medium (Thermo Fisher) supplemented with 25 mM or 5 mM glucose, 100 IU/mL penicillin (Lonza), 100 mg/mL streptomycin (Lonza), B27 supplement (Invitrogen), 2 mM L-glutamine, and 0.3 mM sodium pyruvate for up to 11 days prior to metabolic profiling or XF flux analysis.

### Human induced neural progenitor cell lines

Experiments were carried out using fibroblasts taken from punch biopsy obtained from up to three *C9orf72* G4C2-repeat-positive ALS cases and three age and sex-matched controls. All lines used are shown in Supplementary Table 8. The average age at time of skin biopsy in ALS cases was 54 (SD ± 9.0) years and in controls was 52 (SD ± 12.1) years.

Human induced neural progenitor cell (iNPC) and induced astrocyte (iA) culture: Fibroblasts were differentiated as previously described [23]. iNPCs and iAstrocytes were grown on 5 mg/mL fibronectin (R&D Systems) coated 10-cm dishes, at 37 °C with 5% CO_2_ in humid incubators. iNPCs were cultured in DMEM/F-12 + GlutaMAX™ (Gibco 31,331,093), with 1% N2 supplement (Gibco 17,502,001), 1% B27 supplement (Gibco 17,504,001), and 20 ng/mL FGF (Peprotech 100-18B). iNPCs were differentiated into induced astrocytes for 7 days, by media change to DMEM (4.5 g/L glucose, 25 mM L-glutamine, with phenol red; Sigma Aldrich D5796) with 10% foetal bovine serum (Labtech, FCS-SA/500) and 0.3% N2. Assessment of markers of differentiation in the iAstrocytes was performed as previously described [24].

### Liquid chromatography-mass spectrometry analysis

On day 6 of differentiation, cell media was changed to DMEM (no phenol red, glucose, L-glutamine; Gibco A14430-01), supplemented with 10% FBS, 5 mM D-glucose or 5 mM D-fructose and 0.3 nM L-glutamine. On day 7 of differentiation, cell culture media was removed, and plates were washed once with PBS and discarded. One millilitre of − 20 °C methanol was added and evenly spread across the surface of the plate. Cells were scraped into the methanol, and the suspension was collected in pre-weighed 1.5-mL Eppendorf tubes. After collection, the tubes were spun in MiVac evaporating centrifuge (Genevac) at 250 RCF (max speed) at 30 °C with open lids, until all ethanol had evaporated. Cell pellets were then weighed and stored at − 80 °C until analysis. Prior to analysis, the samples were re-suspended in 200 µL of 50:50 methanol:water (LC–MS grade), vortexed for 1 min and then placed in an ultrasonic bath for 10 min. Samples were centrifuged for 5 min at 1200 rpm and 80 µL of the resulting solution was loaded into a HPLC vial and run automatically on a Waters Synapt G2Si high resolution TOF–MS coupled to a Waters Acquity UPLC. The column was a Waters BEH-Amide column (1.7 µm particle size, 2.1 × 150 mm dimensions). The mobile phases consisted of 0.1% formic acid (A) and acetonitrile + 0.1% formic acid (B). The flow rate was 0.4 mL/min and the injection volume was 4 µL. The gradient programme was as follows: 1–40% A (0–5 min), 40–1% A (5–9 min), 1% A (9–11 min) with a total run time of 11 min per sample. Samples were run using electrospray ionisation in negative mode with a capillary voltage of 3.2 V, sampling cone voltage of 64 V, desolvation gas 590 L/h, and a source temperature of 100 °C. Data for the compounds of interest were collected and processed using Waters MassLynx and QuanLynx software. All data were normalised to the total ion current.

### Seahorse XF24 bioanalyser assays

On the day of the assay, the culture medium was replaced with Dulbecco’s modified Eagle medium containing either 25 mM or 5 mM glucose, 1 mM glutamine, and 0.4 mM sodium pyruvate. Glycolytic flux and mitochondrial respiration were assessed under physiological conditions and after adding 1 µM oligomycin, 0.3 µM FCCP (carbonyl cyanide-4-(trifluoromethoxy)phenylhydrazone), and 1 µM rotenone/antimycin A on a Seahorse XF24 bioanalyser (Agilent). The results were normalised to cell number by adding CyQUANT (Invitrogen) to each well in accordance with the manufacturer’s instructions (1/400 dilution of the dye in HBSS buffer, 50 μL per well). Fluorescence was measured using a BMG Omega FLUOstar. For analysis purposes, nontransgenic flux levels were set to 100% and transgenic flux levels were directly compared.

### Phenotypic metabolic array analysis

On the ninth day of post neural plating, 50 mL of Neurobasal-A with B27 and 0.3 mM glutamine were added to each well of PM-M1 (Biolog) plates, which were then incubated at 37 °C and 5% CO_2_ overnight. On day 10, the neuronal medium was removed from the cells and replaced with the medium from the PM-M1 plate. The cells were then incubated for 24 h prior to adding 10 μL Redox dye MB (Biolog). After dye addition, the plates were sealed and incubated in an OmniLog TM Phenotype Microarray system at 37 °C. NADH production was monitored every 5 min for 350 min. The plates were then washed with phosphate-buffered saline and stored at − 80 °C for 24 h prior to adding CyQUANT. All kinetic data were normalised to CyQUANT fluorescence and were analysed as previously described [24]. Briefly, all data (nontransgenic and transgenic *n* = 6) had background values removed, metabolic flexibility was calculated [24], and the relevant substrates underwent kinetic analysis via two-way ANOVA with Sidak posttest correction at every time point.

## Results

### Unbiased MR causally links serum concentration of five metabolites to ALS risk

Hypothesising that plasma metabolites may influence CNS tissue to modify the biological processes leading to ALS, we used a two-sample MR screen of the entire set of circulating metabolites to determine which metabolites are causally related to ALS. To achieve this, we obtained genetic variants associated with the circulating concentration of 575 metabolites in 7284 individuals [9, 10]. Genetic liability to ALS was measured in 29,612 ALS cases and 122,656 controls [7]. In each MR test, metabolite-associated genetic variants were used as genetic instruments for measuring the effect of metabolite exposure on ALS risk. The complete results of our metabolome screen are presented in Supplementary Table 1.

**Fig. 2:**
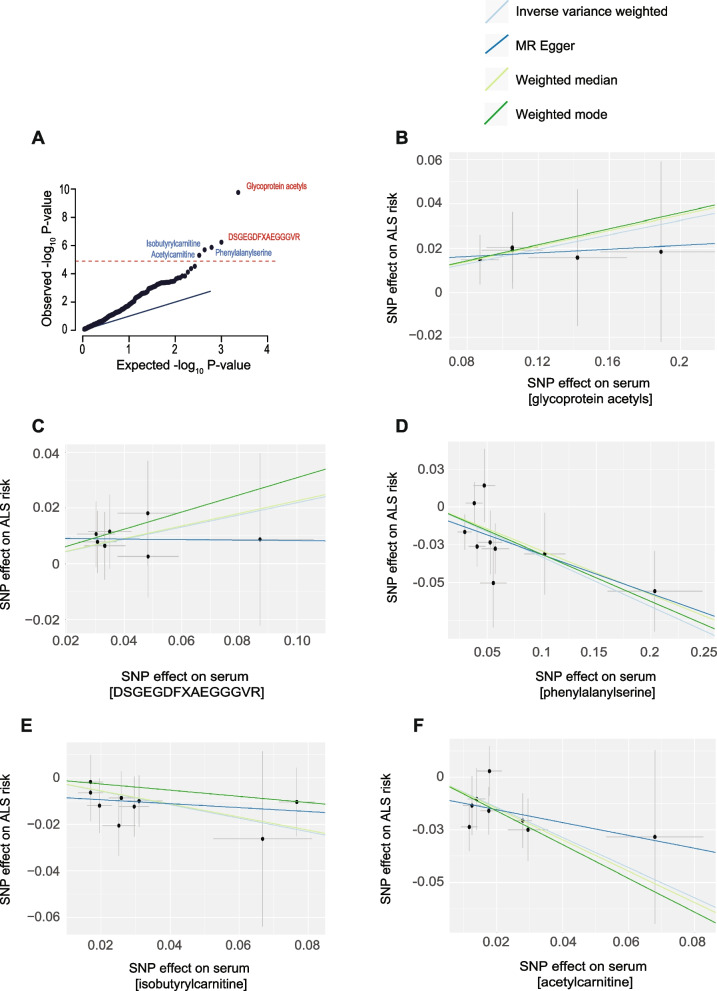
Unbiased two-sample MR screen for circulating metabolites causally linked to ALS risk. **A** Quantile–quantile plot of inverse-variance weighted *P* values demonstrates that five metabolites passed a Bonferroni multiple-testing correction (red line). Blue text represents a protective association, and red text represents a harmful association. Scatter plots demonstrate a significant association of serum metabolite concentrations with ALS risk, including in robust MR tests for glycoprotein acetyls (**B**), DSGEGDFXAEGGGVR (**C**), phenylalanylserine (**D**), isobutyrylcarnitine (**E**), and acetylcarnitine (**F**). Each point represents the effect size (*β*) and standard errors for each SNP–outcome relationship

After a stringent Bonferroni correction, five metabolites were significantly associated with ALS risk. Two metabolites increased ALS risk: glycoprotein acetyls (IVW *P* = 9.7e − 9, *β* = 0.21, *SE* = 0.04) and DSGEGDFXAEGGGVR (IVW *P* = 8.0e − 6, *β* = 0.22, *SE* = 0.05) (Fig. 2A, B, C, Table 1). Notably, these are atypical metabolites: glycoprotein acetyls is a composite measure of a set of glycoproteins linked to the acute phase response [25], and DSGEGDFXAEGGGVR is a matrix-metalloproteinase cleavage product of fibronectin which is indirectly linked to glucose metabolism [26, 27]. Three metabolites were protective against ALS: phenylalanylserine (IVW *P* = 1.6e − 5,* β* = − 0.33, *SE* = 0.08), isobutyrylcarnitine (IVW *P* = 2.0e − 5, *β* = − 0.29, *SE* = 0.07), and acetylcarnitine (IVW *P* = 5.0e − 5, *β* = − 0.72, *SE* = 0.18) (Fig. 2A, D, E, F, Table 1). None of the tests was invalidated by instrument pleiotropy or weak instruments (Table 1).

**Table 1:** Robust MR measures for the effect of significant metabolites on ALS risk

Test	Glycoprotein acetyls	DSGEGDFXAEGGGVR	Phenylalanylserine	Isobutyrylcarnitine	Acetylcarnitine
IVW *P* value	9.7e − 9*	8.0e − 6*	1.6e − 5*	2.0e − 5*	5.0e − 5*
IVW *β*	0.2	0.2	− 0.3	− 0.3	− 0.7
Weighted median *P* value	0.046*	0.2	0.02*	0.09	0.01*
Weighted median *β*	0.2	0.2	− 0.3	− 0.3	− 0.7
Egger *P* value	0.9	1.0	0.1	0.8	0.6
Egger *β*	0.04	− 0.01	− 0.3	− 0.08	− 0.3
Weighted mode *P* value	0.2	0.2	0.04*	0.5	0.06
Weighted mode *β*	0.2	0.3	− 0.3	− 0.1	− 0.8
Mean *F*-statistic	43	22	23	51	36
IVW Cochran’s *Q* test *P* value	0.2	0.8	5.7	2.2	4.0
Radial MR outlier SNPs	2	1	1	1	0
Egger intercept test	0.8	0.6	0.5	0.4	0.5
*I*^2^	0.95	0.94	0.96	0.98	0.97
Number of SNPs LOO > 0.05	0	0	0	0	0
Total number of SNPs	5	7	9	9	8
Number of pleiotropic SNPs	0	0	0	0	0

Radial MR outlier SNPs identified were removed before final model estimation to reduce heterogeneity and improve causal inference accuracy

*IVW* inverse-variance weighted, *MR* Mendelian randomisation, *LOO* leave-one-out analysis, *SNP* single-nucleotide polymorphism

*P < 0.05

Notably, glycoprotein acetyls was also significantly associated with ALS risk in the weighted median estimate (*P* = 0.02, *β* = 0.19). Phenylalanylserine remained significant across multiple robust MR estimates, including the weighted median (*P* = 0.03, *β* = − 0.29) and weighted mode (*P* = 0.04, *β* = − 0.31; Table 1).

### Causally associated metabolites are confirmed with MR in an independent dataset

Two of the five metabolites causally associated with ALS after multiple-testing correction are carnitines. Consequently, we evaluated the protective role of acetylcarnitine and isobutyrylcarnitine using genetic instruments derived from different independent GWAS. Validation increases the likelihood that the identified association is a genuine biological effect and not a false positive [5].

Using four different GWASs of circulating serum/plasma acetylcarnitine measured in different populations including in athletes, we confirmed a protective effect of circulating acetylcarnitine on genetic liability to ALS (IVW *P* < 0.05, *β* < − 0.3, Table 2, Supplementary Fig. 1 A, B, C). Similarly, we confirmed a protective effect of plasma isobutyrylcarnitine on ALS risk (IVW *P* = 0.03, *β* = − 0.04, Table 2, Supplementary Fig. 1D). Overall, MR provided good evidence across multiple populations for a protective effect of circulating carnitines on ALS risk.

**Table 2:** Metabolites causally associated with ALS are confirmed via MR using an independent exposure GWAS to derive genetic instruments

Metabolite	Reference	Sample size	IVW *P* value	Sample type	*β*	Number of SNPs
Acetylcarnitine	[14]	7478 European decent	0.04	Serum	− 0.30	2
	[15]	490 athletes (Qatar, Italy)	5.3e − 41	Serum	− 0.02	2
	[16]	8262 Canadians of European descent	1.02e − 269	Plasma	− 0.08	2
Isobutyrylcarnitine	[16]	8262 Canadians of European descent	0.03	Plasma	− 0.04	5
DSGEGDFXAEGGGVR	[17]	14,296 European decent	0.02	Plasma	0.04	57

In our initial MR screen, serum DSGEGDFXAEGGGVR concentration was linked to increased ALS risk. We obtained an independent GWAS of plasma DSGEGDFXAEGGGVR, in which we confirmed a harmful effect of increased DSGEGDFXAEGGGVR concentration (IVW *P* = 0.02, *β* = + 0.04, Table 2, Supplementary Fig. 1E). No independent GWAS measurements were available for glycoprotein acetyls or phenylalanylserine.

### Direct measurement of carnitines in ALS patient biofluids confirms an association with ALS risk and severity

Instead of a direct measurement, MR utilises genetic liability to an exposure (for example, circulating acetylcarnitine concentration). To confirm a relationship between carnitine availability and ALS pathogenesis, we directly measured the concentration of carnitines in plasma and CSF from patients with ALS and controls.

**Fig. 3:**
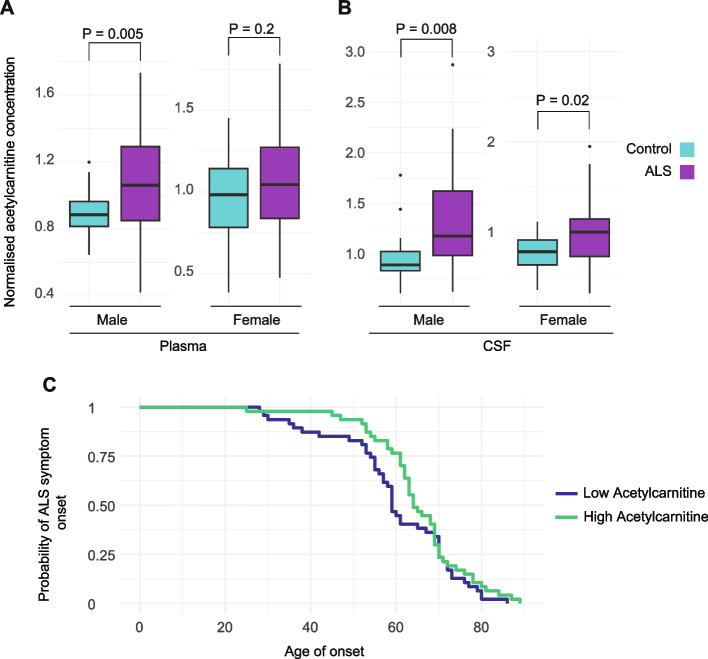
Directly measured acetylcarnitine concentration in patient biofluids is elevated in ALS and associated with delayed symptom onset. **A**, **B** Blood plasma and cerebrospinal fluid (CSF) samples were obtained from 94 patients and 40 controls. Acetylcarnitine concentration in plasma (**A**) and CSF (**B**) separated by sex normalised by the median concentration per sample batch. *P* values determined using unpaired *t*-test. **C** Kaplan–Meier plot of ALS age at symptom onset stratified by plasma acetylcarnitine concentration (high vs low)

Acetylcarnitine concentration was significantly elevated in both plasma (Supplementary Table 2) and CSF from patients with ALS, adjusting for age and stratifying for sex (plasma: ANOVA, male *P* = 0.005, female *P* = 0.2; CSF: male *P* = 0.008, female *P* = 0.02, Fig. 3A, B). Higher plasma acetylcarnitine concentration was significantly associated with later age at onset in plasma ALS samples after adjustment for sex (Cox regression, *P* = 0.02, *HR* = 0.4; Fig. 3C) and this association was particularly evident in males (Supplementary Fig. 2, Supplementary Fig. 3). Similarly, a higher plasma concentration of three other carnitines (3-hydroxyhexanoylcarnitine, 3-hydroxybutyrylcarnitine, and linoleoylcarnitine) was also associated with later age at onset in males after adjustment for multiple testing (*FDR* < 0.05; Supplementary Fig. 2). No association was observed between plasma acetylcarnitine concentration and age at sampling in healthy controls (*P* = 0.6 and *P* = 0.9 for females and males, respectively; Supplementary Fig. 3). There was no significant relationship between plasma or CSF carnitine concentration and ALS patient survival time (Cox regression, *P* > 0.05).

By stratifying for sex, we noted that the association between the directly measured plasma concentration of carnitines, and ALS risk and severity, was particularly evident in males. Similarly, sex-stratified MR confirmed that a higher circulating concentration of acetylcarnitine is causally associated with reduced ALS risk in males (*P* = 0.003, *β* = − 1.13, *SE* = 0.39, Supplementary Fig. 4A) but not in females (*P* = 0.6, *β* = − 0.08, *SE* = 0.1; Supplementary Fig. 4B).

Carnitines are important for the transfer of fatty acids across the inner mitochondrial membrane, which is a key step in β-oxidation for energy production [28]. Neurons favour oxidative phosphorylation of glucose over fatty acid oxidation for energy production [29]. Unlike neurons, microglia, or oligodendrocytes; astrocytes perform high levels of fatty acid oxidation for energy production [30]. Our results suggest that increased plasma concentration of carnitines, which should facilitate energy production by astrocytes, ameliorates neurotoxicity in ALS leading to delayed symptom onset.

### Direct measurement of simple carbohydrates in ALS patient biofluids confirms an association with ALS risk and severity

The hallmark of ALS is progressive loss of MN. Neurons selectively rely on oxidative phosphorylation of glucose for energy production [29]. Under conditions of high energy demand or energetic stress, when glucose uptake via the neuron-specific transporter GLUT3 is insufficient, neurons increase utilisation of lactate supplied from astrocytes [31]. DSGEGDFXAEGGGVR, which we linked to ALS risk in our MR analysis (Fig. 2C), has been linked to neuronal energy metabolism via an effect on function of GLUT3 [32]. We hypothesised that direct supply of energy substrate to MN, including glucose and lactate, would modify ALS risk and severity; therefore, we directly measured the concentration of simple carbohydrates in plasma and CSF from patients with ALS and controls (Supplementary Table 3). MN can utilise both pyruvate and mannose but do so minimally because the concentration of both is relatively low in CNS interstitial fluid. We also measured fructose, another simple carbohydrate, which is poorly utilised by neurons due to the relative absence of GLUT5 which is necessary for intracellular uptake; in contrast, fructose is significantly utilised by glia cells including astrocytes and microglia [33–35].

**Fig. 4:**
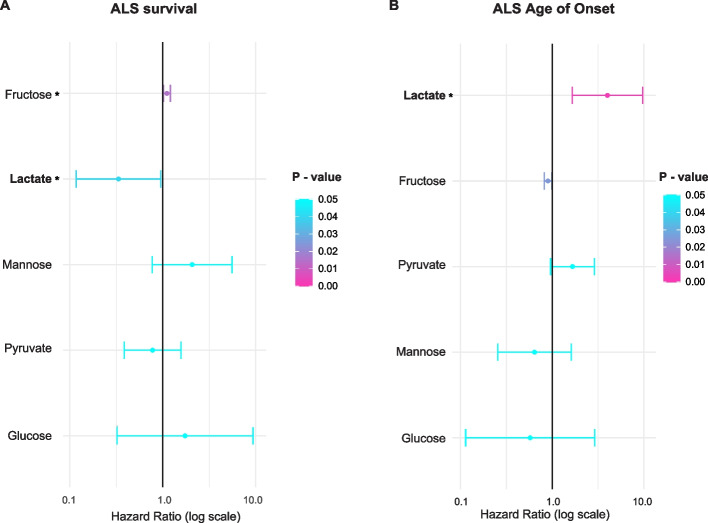
Directly measured concentrations of simple carbohydrates in plasma are associated with ALS survival and age at symptom onset. **A** Multivariate Cox regression results for the association between plasma concentration of simple carbohydrates and time from ALS diagnosis until death (i.e. survival). **B** Multivariate Cox regression results for the association between plasma concentration of simple carbohydrates and age at ALS diagnosis. *P < 0.05

Both glucose and lactate are utilised directly by neurons including MN. Plasma concentration of glucose is not associated with ALS age of onset or survival (Fig. 4A, B). In contrast, higher plasma lactate concentration is associated with longer ALS patient survival (Cox regression, *P* = 0.03, *HR* = 0.33; Fig. 4A, Supplementary Table 4). Higher plasma lactate concentration is also significantly associated with earlier age of ALS symptom onset (Cox regression, *P* = 0.01, *HR* = 3.07; Fig. 4B, Supplementary Table 5). Lactate was elevated in ALS patient CSF compared to controls (ANOVA, *P* = 1.3e − 3, Supplementary Fig. 5); there was also a non-significant trend towards an increase in lactate concentration in ALS patient plasma (Supplementary Fig. 5). We interpret our findings to indicate that lactate is important for disease progression but not onset and, in that context, we suggest that elevated lactate is a response to disease onset rather than a cause of earlier disease onset.

The plasma concentrations of pyruvate and mannose were not associated with ALS age of onset or survival (Fig. 4A, B). Higher plasma fructose concentration is associated with shorter ALS patient survival (Cox regression, *P* = 0.015, *HR* = 1.1; Fig. 4A, Supplementary Table 4).

Our MR analysis found no evidence that genetically determined circulating concentration of fructose or lactate influences ALS risk suggesting that utilisation of fructose and lactate could have a selective effect on disease progression.

### Fine-mapping of SNPs linked to plasma DSGEGDFXAEGGGVR concentration implicates impaired neuronal glucose uptake in ALS pathogenesis

The plasma concentration of DSGEGDFXAEGGGVR, a product of matrix-metalloproteinase cleavage of fibronectin, is significantly associated with SLC2A3 gene expression [32]. *SLC2A3* encodes GLUT3, the primary glucose uptake transporter in neurons. Of the genetic instruments we used to assess DSGEGDFXAEGGGVR concentration, the rs10846162 ‘C’ allele was linked to a higher plasma DSGEGDFXAEGGGVR concentration (*P* = 1.3e − 5, *β* = + 0.04, *SE* = 9.2e − 3) and higher ALS risk (ALS GWAS *P* = 0.004, *β* = + 0.04). Notably, this SNP is also an expression quantitative trait locus (eQTL) for GLUT3 expression (*n* = 5229, *P* = 1.3e − 4, normalised effect size (*NES*) = 0.1) in arterial endothelium. We have confirmed the validity of this eQTL in iPSC-derived MN sourced from patients with ALS (*n* = 210 ALS patients/lines; *P* = 0.01; *R* = + 0.15; logistic regression with age, sex, and the first 10 principal components (PCs) as covariates). Moreover, MR using rs10846162 demonstrated a significant link between increased plasma DSGEGDFXAEGGGVR, higher SLC2A3 expression, and increased ALS risk (Wald ratio *P* = 4.0e − 3, *β* = 1.02, *SE* = 9.0e − 3). The rs10846162 ‘C’ allele is also associated with spinal as opposed to bulbar onset ALS (*P* = 0.03; *β* = 0.1; *SE* = 0.05; logistic regression with age, sex, and 1–10 PCs as covariates).

### Energy production is impaired in neurons carrying an ALS-associated C9orf72 mutation

We hypothesised that ALS might occur because of a synergistic interaction whereby reduced energy substrate availability coincides with a selective vulnerability of ALS patient MN. To study this, we investigated the metabolic flexibility of neurons carrying an ALS-associated G4C2-repeat expansion of *C9orf72* [8], which is the most common genetic risk factor for ALS [36]. Cortical neurons were isolated from mice with knock-in of a mutated BAC containing a G4C2-repeat expansion of *C9orf72* [8] and healthy control mice. These neurons were cultured under conditions of varying glucose concentrations to induce a state of glucose deprivation. This approach does not model increased exogenous lactate availability in vivo (e.g. astrocyte/neuron coupling [31]), but instead tests neuronal energetics without supplemental lactate, minimising masking by extracellular lactate and requiring reliance on endogenous substrate handling; we propose that this is particularly relevant in ALS where astrocyte function is impaired [24]. Our data presented above linked plasma and CSF lactate concentration to ALS severity (Fig. 4A, B).

**Fig. 5:**
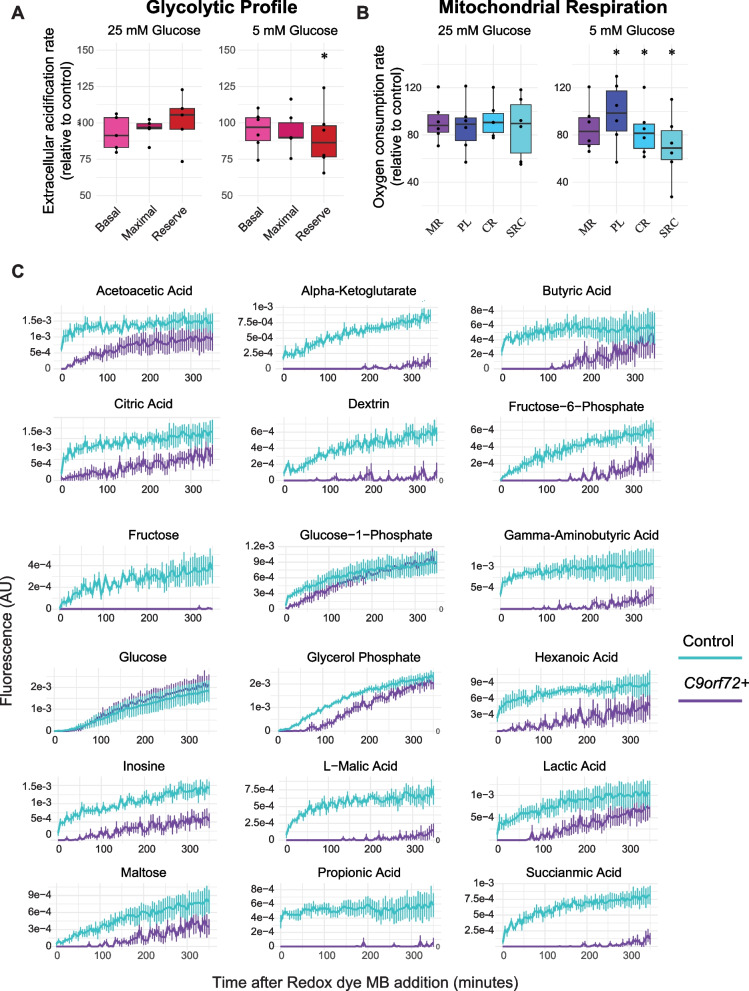
Energetic flexibility of mouse cortical neurons is impaired in the presence of a G4C2-repeat expansion of *C9orf72*. **A** Seahorse XF24 analysis of mitochondrial respiration in mouse cortical neurons cultured with either 5 mM or 25 mM glucose. Basal oxygen consumption rate (left), maximal respiratory capacity (middle), and reserve respiratory capacity (right) were measured by extracellular acidification rate under physiological conditions and under low glucose conditions. Values are for *C9orf72* + neurons standardised to control neurons. **B** Seahorse XF24 analysis of glycolytic profile in *C9orf72* + neurons cultured with either 5 mM or 25 mM glucose. Coupled respiration (CR), maximal respiratory capacity (MR), proton leak (PL), and spare respiratory capacity (SRC) were measured by oxygen consumption rate. **C** Phenotypic metabolic array analysis for *C9orf72* + and control neurons. NADH production was measured every 5 min for 350 min following the addition of Redox dye MB. Data are plotted as minutes elapsed after dye addition on the x-axis, and values were normalised to cell number

Metabolic function was assessed using a Seahorse XF24 analyser, which provided measurements of both glycolysis and mitochondrial respiration (Fig. 5A, B and Additional file 1: Tables 1–4). Under conditions of reduced glucose, cortical neurons expressing a *C9orf72* G4C2-repeat expansion (*C9orf72* +) displayed a significant impairment in mitochondrial function compared with control neurons. Specifically, under conditions of reduced glucose (but not normal glucose), *C9orf72* + neurons demonstrated lower glycolytic reserve capacity (Wilcox test, *P* < 0.05, Fig. 5A) and diminished coupled respiration, increased proton leak, and reduced spare respiratory capacity (Wilcox test, *P* < 0.05, Fig. 5B).

### C9orf72 + neurons and astrocytes are impaired in their ability to utilise alternative carbon substrates

We hypothesised that *C9orf72* + neurons might have impaired mitochondrial function because they are unable to utilise diverse carbon substrates which would make them selectively vulnerable to deficiency of energy substrate. To test this, we measured NADH production as an indicator of energy substrate metabolism under conditions of glucose deprivation. After supplying alternative substrates including carbohydrates, carboxylic acids (including lactate), and fatty acids, *C9orf72* + neurons produced significantly less NADH than controls (repeated measures ANOVA, *P* < 0.05, Fig. 5C, Supplementary Table 6, Additional file 1: Tables 5–22). Consistent with this finding, *C9orf72* + neurons had significantly lower metabolic flexibility index (ANOVA, *P* = 0.04) [24], confirming the reduced ability to switch to alternative energy sources during stress such as glucose deprivation. This impaired flexibility may explain why ALS risk and severity is linked to energy substrate availability.

We have previously observed that ALS astrocytes also have impaired metabolic flexibility [24]. Direct measurement of plasma simple carbohydrates revealed that higher concentration of fructose is linked to shorter ALS survival (Fig. 4A, Supplementary Table 4). Fructose is particularly used for energy production by astrocytes and microglia [33–35]. Previously, we demonstrated that ALS patient-derived astrocytes have defects in fructose metabolism including reduced production of NADH from fructose compared to control-derived astrocytes, which was not due to loss of GLUT5 or glycolytically linked fructose metabolism enzymes [24]. To expand upon this observation, we examined the ability of *C9orf72* + patient-derived astrocytes to metabolise fructose for energy production: many downstream glycolytic and TCA cycle products of fructose metabolism were significantly deficient in *C9orf72* + astrocytes supplied with fructose as an energy source compared to control astrocytes (ANOVA, *P* < 0.05, Supplementary Fig. 6) consistent with a disease-associated deficiency in astrocyte fructose metabolism.

## Discussion

Inadequate energy production has long been associated with neurodegenerative disease [37–39], but questions have remained regarding the position of energy deficits in the pathophysiological cascade [40] and cell specificity [41]. TDP-43 mislocalisation within MN, which is the hallmark of > 97% of ALS cases [42], is associated with TDP-43 entry into the mitochondria and impaired bioenergetic function [43]. We have previously causally linked mitochondrial function to ALS survival [44]. In the current study, we provide evidence for a causal link between availability of energy substrate to multiple disease-relevant CNS cell types and both ALS risk and severity. We have defined ALS severity to include both earlier age of symptom onset and shorter survival time.

We provide multiple lines of evidence for the *protective* role of the circulating carnitines against onset of ALS, particularly in males. We applied MR in multiple populations using distinct genetic instruments, and we performed a direct measurement of carnitine concentration in patient plasma and CSF. Our finding that carnitines are elevated in ALS patient biofluids compared to controls might appear to contradict our finding that circulating carnitines are protective against ALS risk. We interpret our data to suggest that carnitines are beneficial and thus elevation in disease is a compensatory effect. This idea is supported by a recent longitudinal analysis which showed that plasma concentration of carnitines is negatively correlated with disease severity at the time of measurement across multiple time points, but is not significantly related to the rate of disease progression [37]. Similarly, we identified a correlation between plasma and CSF carnitine concentration and ALS age of onset but not survival. We note an ongoing clinical trial is exploring the therapeutic potential of carnitine derivatives [45].

Carnitines are important for the transfer of fatty acids across the inner mitochondrial membrane, which is a key step in β-oxidation for energy production [28]. Neurons favour oxidative phosphorylation of glucose over fatty acid oxidation for energy production [29] although in conditions of high energetic stress a byproduct of neuronal energy production is high levels of toxic peroxidated fatty acids [46]. In contrast, astrocytes perform high levels of fatty acid oxidation for energy production [30] and even process toxic peroxidated fatty acids produced by neurons [46]. We hypothesise but we have not demonstrated conclusively that our observations with respect to carnitines reflect a protective role for astrocyte function. We note a study of a different disease caused by an autoimmune response targeting an astrocyte protein—neuromyelitis optica spectrum disorders (NMOSD)—is also associated with changes in circulating acetylcarnitine [47]. A recent single-cell RNA-sequencing study of ALS motor cortex provides additional evidence for astrocyte dysfunction in ALS: this study identified astrocyte-specific changes in gene expression suggesting impaired support for neurons including both reduced supply of lactate and reduced processing of toxic fatty acids [48]. Our results suggested that carnitines are neuroprotective particularly in males and we note reports of sex differences in astrocyte bioenergetics [49] and the observation that male rat astrocytes have a higher maximal respiration than female astrocytes [49].

Two carnitines associated with a later age of onset which passed FDR multiple-testing correction in males were hydroxylated species, a class of metabolites often interpreted as readouts of mitochondrial fatty acid handling [50]. Hydroxy-acylcarnitines can accumulate when flux through β-oxidation is constrained or when mitochondrial lipid supply exceeds oxidative capacity, promoting diversion of fatty acyl groups onto carnitine as a buffering/export mechanism [51, 52]. Our data suggest this buffering capacity may delay the onset of ALS symptoms perhaps highlighting the role of astrocytes in detoxifying peroxidated fatty acids [46].

In contrast to astrocytes, neurons primarily utilise oxidative phosphorylation of glucose—and other simple carbohydrates such as lactate and pyruvate—for energy production. In particular, lactate is utilised by MN under conditions of stress [53] when uptake of glucose via GLUT3 is insufficient [31]. A higher circulating blood concentration of the peptide DSGEGDFXAEGGGVR, a matrix-metalloproteinase cleavage product of fibronectin, is causally associated with increased ALS risk in our MR analysis. The phosphorylated precursor ADpSGEGDFXAEGGGVR has also been associated with increased ALS risk [54]. These observations are likely linked to energy metabolism within neurons: Circulating DSGEGDFXAEGGGVR concentration has been associated with type 2 diabetes mellitus [26] and responds to glucose ingestion [27]. Notably, DSGEGDFXAEGGGVR is genetically associated with higher expression of *SLC2A3*, which encodes the neuronal glucose transporter GLUT3, through rs10846162, which is also associated with higher ALS risk. Direct measurement of the circulating concentration of simple carbohydrates in ALS patients revealed that elevated lactate, but not glucose, mannose, or pyruvate, is associated with longer ALS survival. In contrast, elevated circulating lactate was *not* associated with delayed age of ALS onset, consistent with the idea that lactate is not relevant for the initiation of disease but only once MN have become stressed. We hypothesise that the effect of GLUT3 function and circulating lactate on ALS risk should be co-dependent, but we were not able to demonstrate that in this study.

A negative effect of higher serum DSGEGDFXAEGGGVR could seem contradictory: If ALS neurons are deficient in energy substrates, then it could be assumed that a GLUT3-mediated increase in glucose transport into neurons would help combat the onset of disease. However, there is good evidence that glucose uptake by neurons can inhibit other forms of energy metabolism, leading to reduced metabolic flexibility [55–57]. We hypothesise that rs10846162 carriers with elevated GLUT3 expression are selectively vulnerable to ALS because their MN are unable to utilise diverse energy substrates under conditions of inadequate energy substrate. Glucose uptake might therefore contribute to the reduced metabolic flexibility we observed in vitro in *C9orf72* + neurons although we have not demonstrated that in this study. We also linked GLUT3 expression not only to ALS risk, but also to the site of symptom onset, which suggests that neuronal glucose uptake capacity may modify selective vulnerability of specific MN subtypes. We note that a previous mouse study of an ALS-associated SOD1 mutation describing a disease-associated shift away from glucose metabolism towards fatty acid oxidation [58] which might be consistent with a requirement for metabolic flexibility.

Fructose is poorly utilised as an energy substrate by neurons due to the relative absence of GLUT5 which is necessary for intracellular uptake; in contrast, fructose is significantly utilised by glia cells including astrocytes and microglia [33–35]. We associated higher plasma fructose concentration with shorter ALS survival time. This suggests a deleterious effect which is not consistent with our other data pertaining to astrocytes. Consistent with this, our in vitro data suggest that ALS patient-derived astrocytes are actually deficient in their ability to utilise fructose. We suggest instead that the harmful effect of fructose might be mediated via a positive effect on proinflammatory microglia. Modulation of proinflammatory microglia has recently been highlighted as a therapeutic strategy in ALS [59].

Our study has several limitations. First, the two-sample MR design relies on the assumption that the genetic instruments influence ALS risk solely through their effect on the metabolite exposures, which cannot be conclusively demonstrated; undetected pleiotropy or residual confounding could bias our causal inferences. For example, a higher circulating concentration of acetylcarnitine [60] could reflect more complete fat oxidation which may reflect healthier mitochondria rather than energy substrate availability. Or the association of isobutyrylcarnitine with reduced ALS risk could relate to the fact that this is a breakdown produce of a branched-chain amino acid which have previously been linked positively to risk of ALS [6]. However, we have attempted to mitigate this possibility via a set of orthogonal verifications which are consistent with our overall hypothesis. Our direct measurements of circulating metabolites were made at a single time point which prohibited a longitudinal analysis that could have supported our suppositions regarding relationships between particular metabolites and disease onset or progression. Our study populations were predominantly of European ancestry, so it is uncertain whether our results apply equally to other ethnic groups or wider populations. Our in vitro study of astrocytes and neurons is limited to cells carrying an ALS-associated C9orf72 expansion, and the neurons were derived from mice not humans. We hypothesise that, given the pathological and phenotypic overlap of *C9orf72* + ALS with the majority of ALS patients [36], these findings will be generalisable but we have not explicitly demonstrated this. Additionally, we have not been able to use vitro co-culture of motor neurons, astrocytes, and microglial cells, so the exact response of motor neurons to starvation in vivo is not fully modelled.

## Conclusions

In conclusion, our multi-layered analysis provides convergent evidence that ALS risk and progression are influenced by metabolic factors, particularly the availability and utilisation of energy substrates by multiple CNS cell types. These findings underscore the importance of energy metabolism in ALS pathophysiology and further highlight that enhancing metabolic flexibility may be a novel therapeutic avenue [61]. Indeed, our results suggest that metabolic interventions, such as dietary supplementation (for example, with carnitines) or strategies targeting neuronal glucose/lactate uptake, could potentially delay disease onset or progression in genetically at-risk individuals.

## Supplementary Information

Additional file 1. Raw data for measurements of NADH production by mouse cortical neurons with and without an ALS-associated *C9orf72* G4C2-repeat expansion in different energy substrates. Tables 1-4 Raw data for Seahorse experiments shown in Fig. 5 A and B. Tables 5–22 Raw data for metabolite utilisation experiment shown in Fig. 5 C and Supplementary Table 6. NTG: nontransgenic control line, TG: transgenic line.

Additional file 2: Supplementary Table 1. Metabolome-wide two-sample Mendelian randomisationscreen for circulating metabolites causally linked to ALS risk. *N* = number of genetic instruments; *SE* = standard error; *Q* = Cochran’s *Q* test; LOO = leave-one-out test. Supplementary Table 2 Comparison of directly measured concentration of carnitines in plasma from ALS patients and controls. Supplementary Table 3 Comparison of directly measured concentration of selected simple carbohydrates in plasma from ALS patients and controls. Supplementary Table 4 Cox regression for the relationship between plasma concentration of selected simple carbohydrates and ALS survival time. Supplementary Table 5 Cox regression for the relationship between plasma concentration of selected simple carbohydrates and ALS age of onset. Supplementary Table 6 NADH production by mouse cortical neurons with and without an ALS-associated *C9orf72* G4C2-repeat expansion in different energy substrates. *P* value for repeated measures ANOVA shown for each energy substrate. Supplementary Table 7 Metadata for ALS patients and controls donating biofluids for direct measurement of metabolite concentrations. Supplementary Table 8 Metadata for ALS patients and controls donating fibroblasts for derivation of iAstrocytes.

Additional file 3: Supplementary Fig. 1. Metabolites causally associated with ALS are confirmed with MR in an independent dataset. Scatter plots demonstrate a significant association of circulating serum/plasma metabolite concentrations with ALS risk via the inverse-variance weighted test for acetylcarnitine, isobutyrylcarnitine, and DSGEGDFXAEGGGVR. Each point represents the effect sizeand standard errors for each SNP–outcome relationship.

Additional file 4: Supplementary Fig. 2. Sex-stratified Cox regression for the association of circulating carnitine concentration on age of ALS symptom onset.Hazard ratios in males for carnitines with *P* < 0.05.Hazard ratios in females for carnitines with *P* < 0.05. Hazard ratios are plotted on a log scale. *P < 0.05; **P < 0.01; †*FDR* < 0.05.

Additional file 5: Supplementary Fig. 3. Sex-stratified Pearson correlations between circulating carnitine concentration and age of ALS symptom onset. Data for females in purple and males in blue. Shown are data for acetylcarnitineand adipoylcarnitine, and also shown is the mean expression value of all measured carnitine metabolites.

Additional file 6: Supplementary Fig. 4. Sex-specific two-sample MR to determine whether there is a causal relationship between circulating concentration of acetylcarnitine and risk of ALS. Scatter plots formales andfemales. Each point represents the effect sizeand standard errors for each SNP–outcome relationship.

Additional file 7: Supplementary Fig. 5. Comparison of lactate concentration in ALS patient and control biosamples. **P < 0.01.

Additional file 8: Supplementary Fig. 6. Fructose metabolism is deficient in *C9orf72* + astrocytes. The effect of fructose supplementation on control and *C9of72* + iAstrocytes metabolite levels by LC–MS analysis. Three control and three *C9orf72* lines were analysed in triplicate. Significance determined via Welch’s *t*-test. *P < 0.05; **P < 0.01.

## Data Availability

The raw data for the metabolic flux experiments can be found in Additional Data 1. Anonymised metabolic profiling data from the AMBRoSIA project can be provided upon request by a qualified researcher.

## Abbreviations

ALS: Amyotrophic lateral sclerosis
MR: Mendelian randomisation
GWAS: Genome-wide association study
ANOVA: Analysis of variance
HR: Hazard ratio
MN: Motor neuron
NMR: Nuclear magnetic resonance
SNP: Single-nucleotide polymorphism
MAF: Minor allele frequency
IVW: Inverse-variance weighted
LOO: Leave-one out
FDR: False discovery rate
BAC: Bacterial artificial chromosome
SD: Standard deviation
NPC: Neural progenitor cell
iAstrocyte: Induced astrocyte
FBS: Foetal bovine serum
RCF: Relative centrifugal force
LC-MS: Liquid chromatography-mass spectrometry
HPLC: High-performance liquid chromatography
TOF-MS: Time-of-flight mass spectrometry
UPLC: Ultra-pure liquid chromatography
HBSS: Hanks’ balanced salt solution
NADH: Nicotinamide adenine dinucleotide
SE: Standard error
CNS: Central nervous system
TCA: Tricarboxylic acid cycle
