# Supplementary material for: Hypothesis-free evaluation of circulating metabolome provides cell-specific insights regarding the role of energy substrate availability in amyotrophic lateral sclerosis
Source: BMC Med. 2026 Mar 6;24:233. doi: 10.1186/s12916-026-04727-w (PMC13077999; doi:10.1186/s12916-026-04727-w)

Supplementary Figure 1: Metabolites causally associated with ALS are confirmed with MR in an independent dataset.

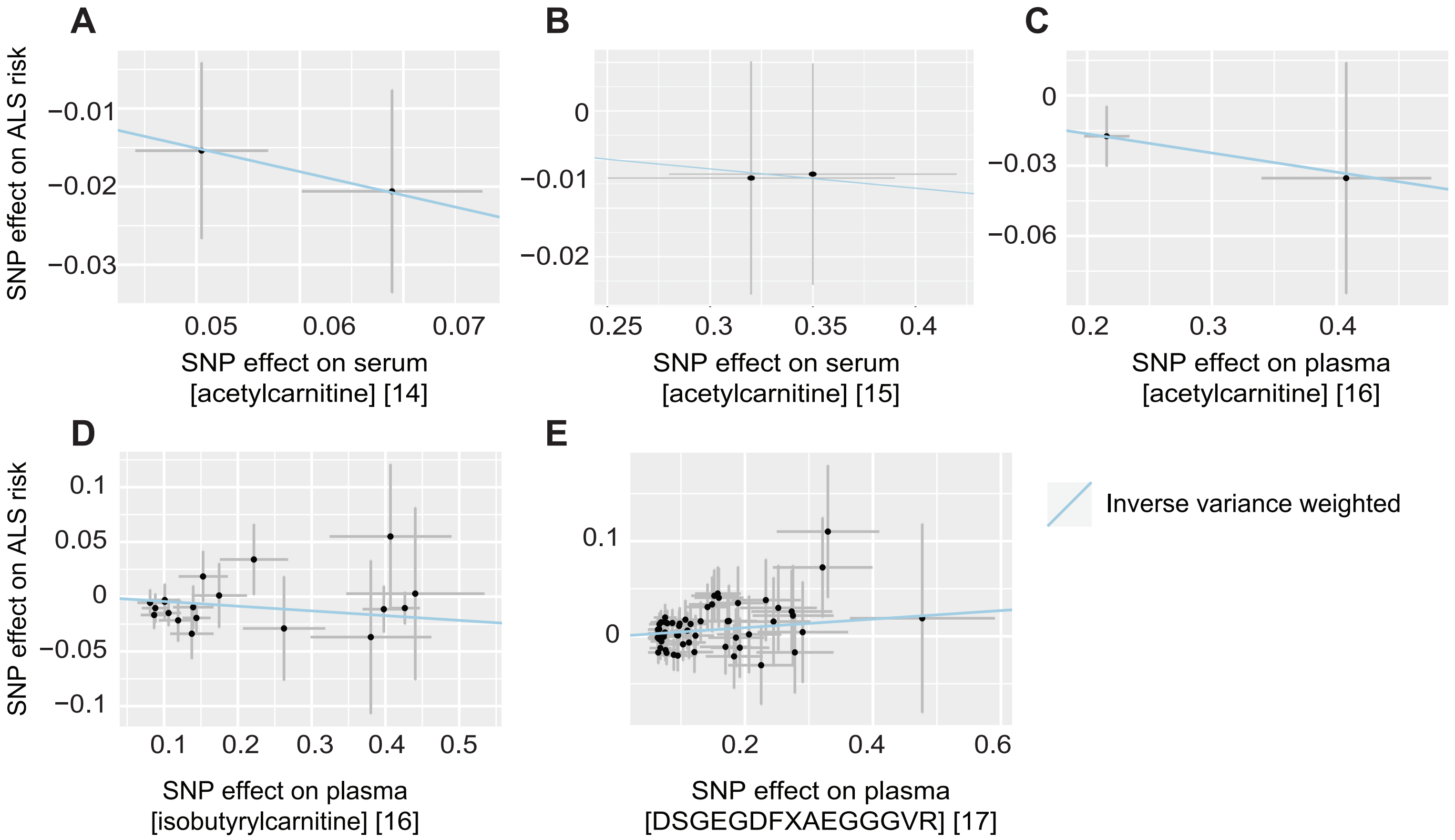

Supplement: Supplementary file 3 — Additional file 3: Supplementary Fig. 1. Metabolites causally associated with ALS are confirmed with MR in an independent dataset. Scatter plots demonstrate a significant association of circulating serum/plasma metabolite concentrations with ALS risk via the inverse-variance weighted test for acetylcarnitine, isobutyrylcarnitine, and DSGEGDFXAEGGGVR. Each point represents the effect sizeand standard errors for each SNP–outcome relationship. [file 12916_2026_4727_MOESM3_ESM.pdf]
