# Supplementary material for: Hypothesis-free evaluation of circulating metabolome provides cell-specific insights regarding the role of energy substrate availability in amyotrophic lateral sclerosis
Source: BMC Med. 2026 Mar 6;24:233. doi: 10.1186/s12916-026-04727-w (PMC13077999; doi:10.1186/s12916-026-04727-w)

Supplementary Figure 2: Sex-stratified Cox regression for the association of circulating carnitine concentration on age of ALS symptom onset.

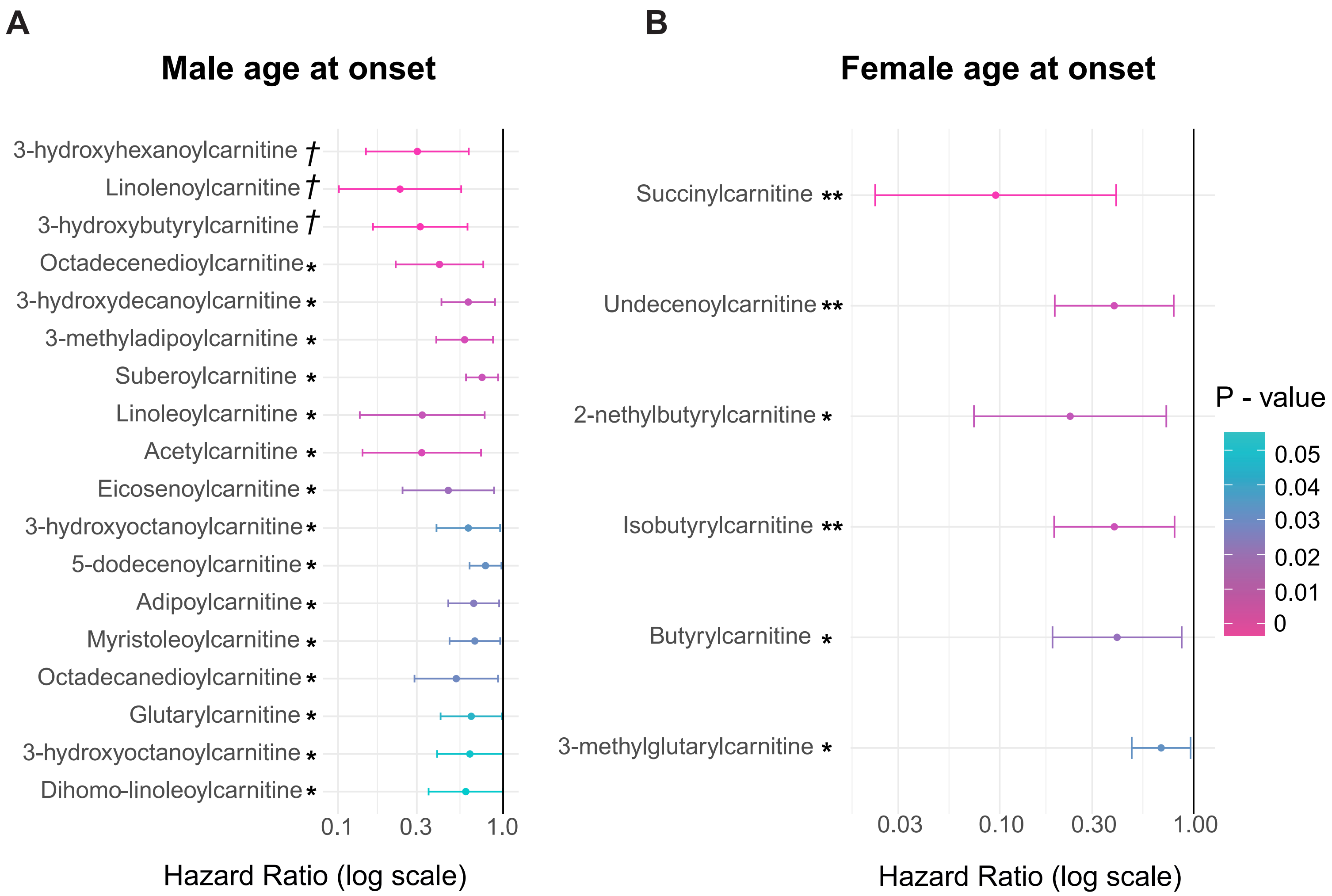

Supplement: Supplementary file 4 — Additional file 4: Supplementary Fig. 2. Sex-stratified Cox regression for the association of circulating carnitine concentration on age of ALS symptom onset.Hazard ratios in males for carnitines with P < 0.05.Hazard ratios in females for carnitines with P < 0.05. Hazard ratios are plotted on a log scale. *P < 0.05; **P < 0.01; †FDR < 0.05. [file 12916_2026_4727_MOESM4_ESM.pdf]
