# Supplementary material for: Hypothesis-free evaluation of circulating metabolome provides cell-specific insights regarding the role of energy substrate availability in amyotrophic lateral sclerosis
Source: BMC Med. 2026 Mar 6;24:233. doi: 10.1186/s12916-026-04727-w (PMC13077999; doi:10.1186/s12916-026-04727-w)

Supplementary Figure 3: Sex-stratified Pearson correlations between circulating carnitine concentration and age of ALS symptom onset.

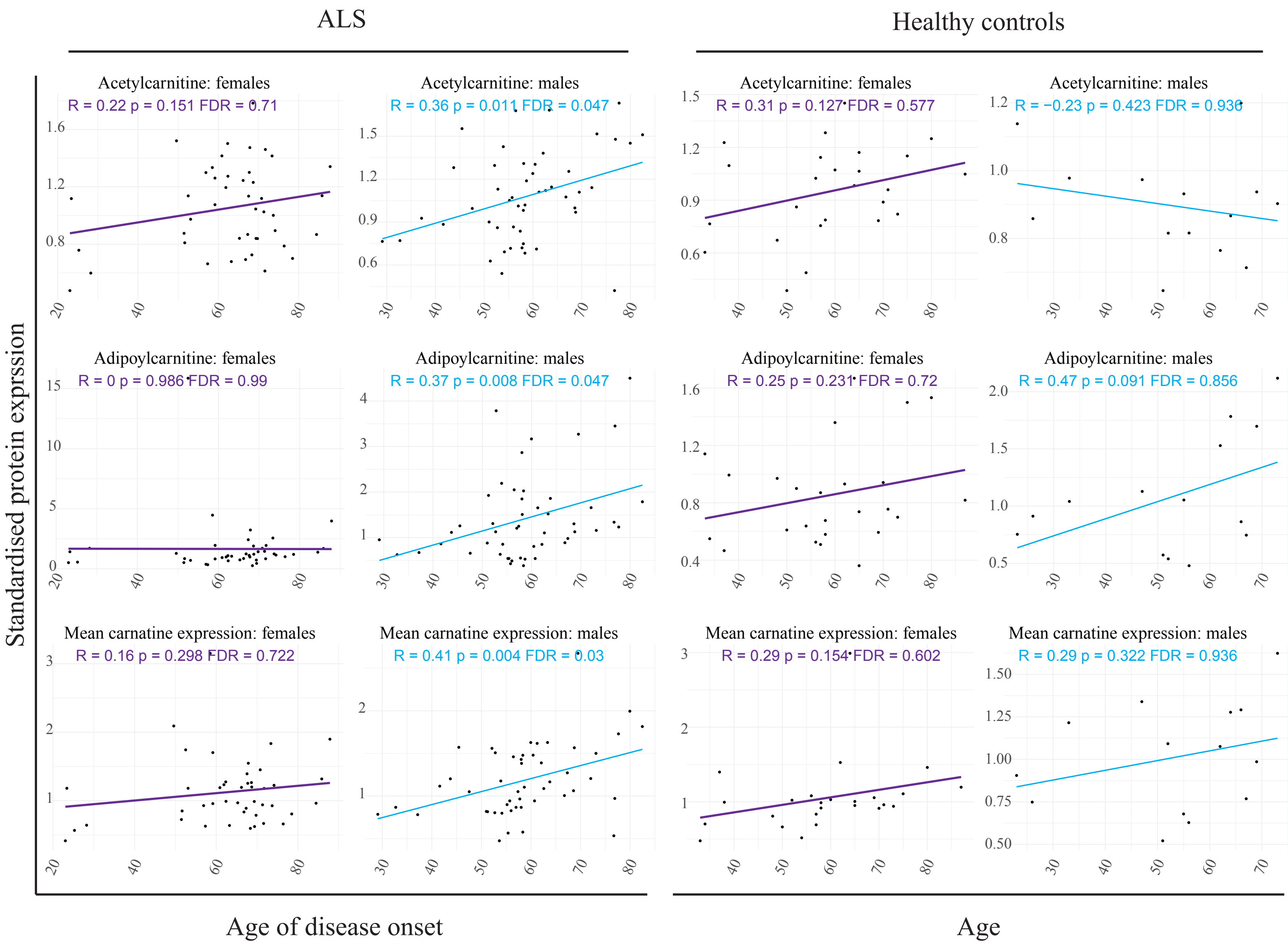

Supplement: Supplementary file 5 — Additional file 5: Supplementary Fig. 3. Sex-stratified Pearson correlations between circulating carnitine concentration and age of ALS symptom onset. Data for females in purple and males in blue. Shown are data for acetylcarnitineand adipoylcarnitine, and also shown is the mean expression value of all measured carnitine metabolites. [file 12916_2026_4727_MOESM5_ESM.pdf]
