# Supplementary material for: Hypothesis-free evaluation of circulating metabolome provides cell-specific insights regarding the role of energy substrate availability in amyotrophic lateral sclerosis
Source: BMC Med. 2026 Mar 6;24:233. doi: 10.1186/s12916-026-04727-w (PMC13077999; doi:10.1186/s12916-026-04727-w)

Supplementary Figure 4: Sex-specific two-sample MR to determine whether there is a causal relationship between circulating concentration of acetylcarnitine and risk of ALS.

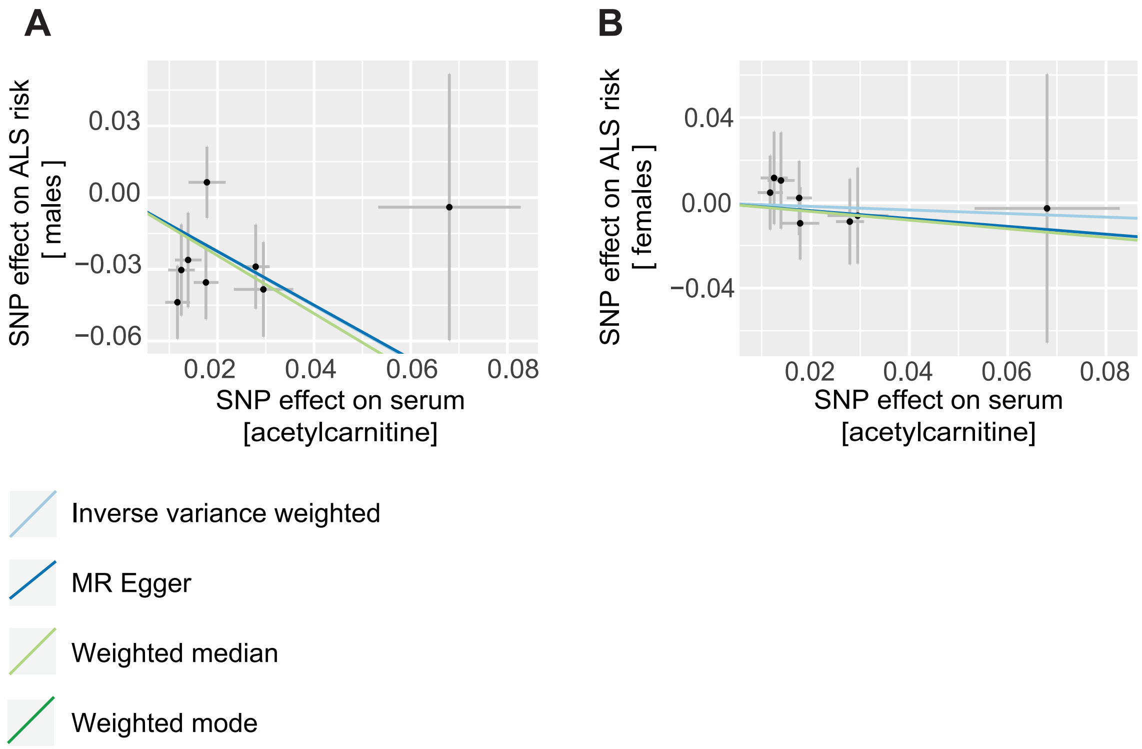

Supplement: Supplementary file 6 — Additional file 6: Supplementary Fig. 4. Sex-specific two-sample MR to determine whether there is a causal relationship between circulating concentration of acetylcarnitine and risk of ALS. Scatter plots formales andfemales. Each point represents the effect sizeand standard errors for each SNP–outcome relationship. [file 12916_2026_4727_MOESM6_ESM.pdf]
