# Supplementary material for: Hypothesis-free evaluation of circulating metabolome provides cell-specific insights regarding the role of energy substrate availability in amyotrophic lateral sclerosis
Source: BMC Med. 2026 Mar 6;24:233. doi: 10.1186/s12916-026-04727-w (PMC13077999; doi:10.1186/s12916-026-04727-w)

Supplementary Figure 5: Comparison of lactate concentration in ALS patient and control biosamples.

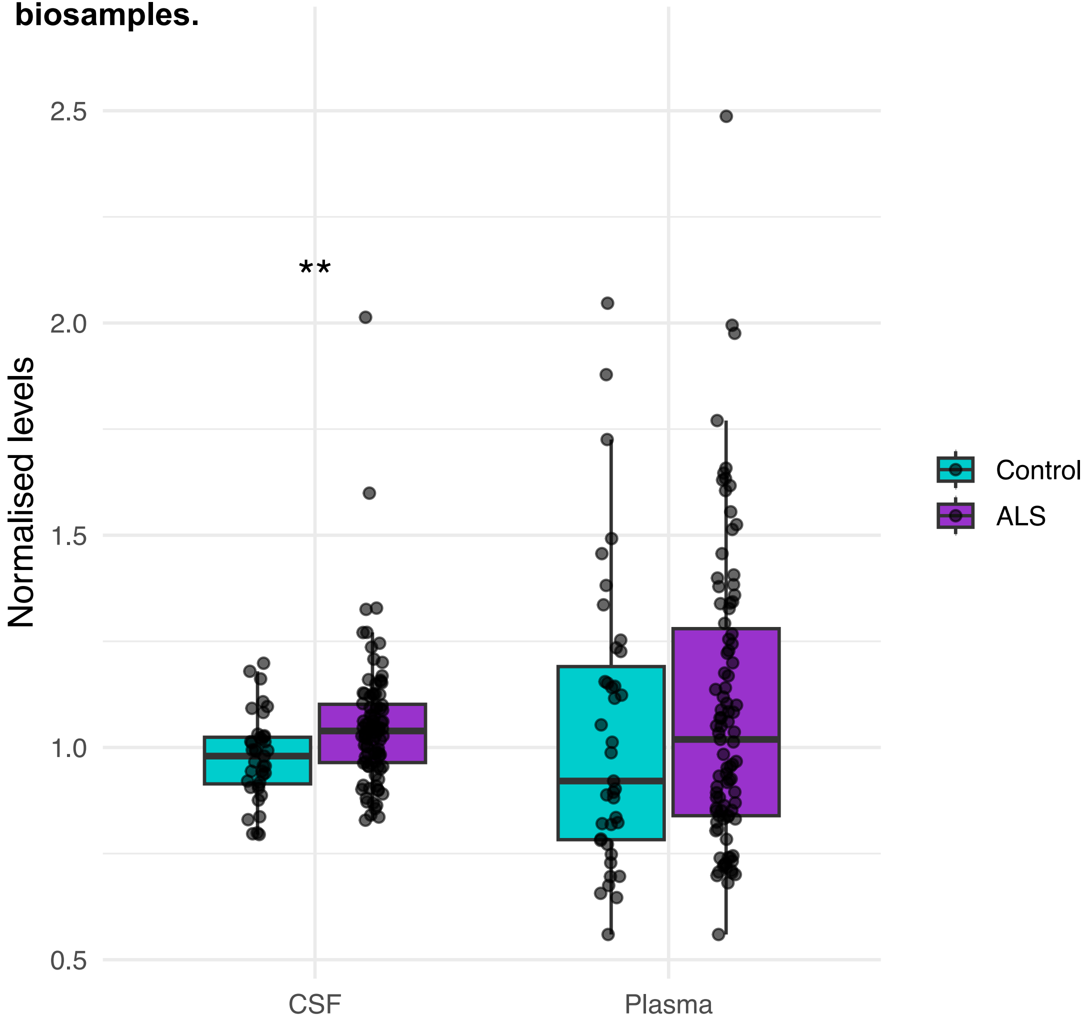

Supplement: Supplementary file 7 — Additional file 7: Supplementary Fig. 5. Comparison of lactate concentration in ALS patient and control biosamples. **P < 0.01. [file 12916_2026_4727_MOESM7_ESM.pdf]
