# Supplementary figures and images for: Hypothesis-free evaluation of circulating metabolome provides cell-specific insights regarding the role of energy substrate availability in amyotrophic lateral sclerosis
Source: BMC Med. 2026 Mar 6;24:233. doi: 10.1186/s12916-026-04727-w (PMC13077999; doi:10.1186/s12916-026-04727-w)

**Supplementary Figure 6: Fructose metabolism is deficient in C9orf72+ astrocytes.**

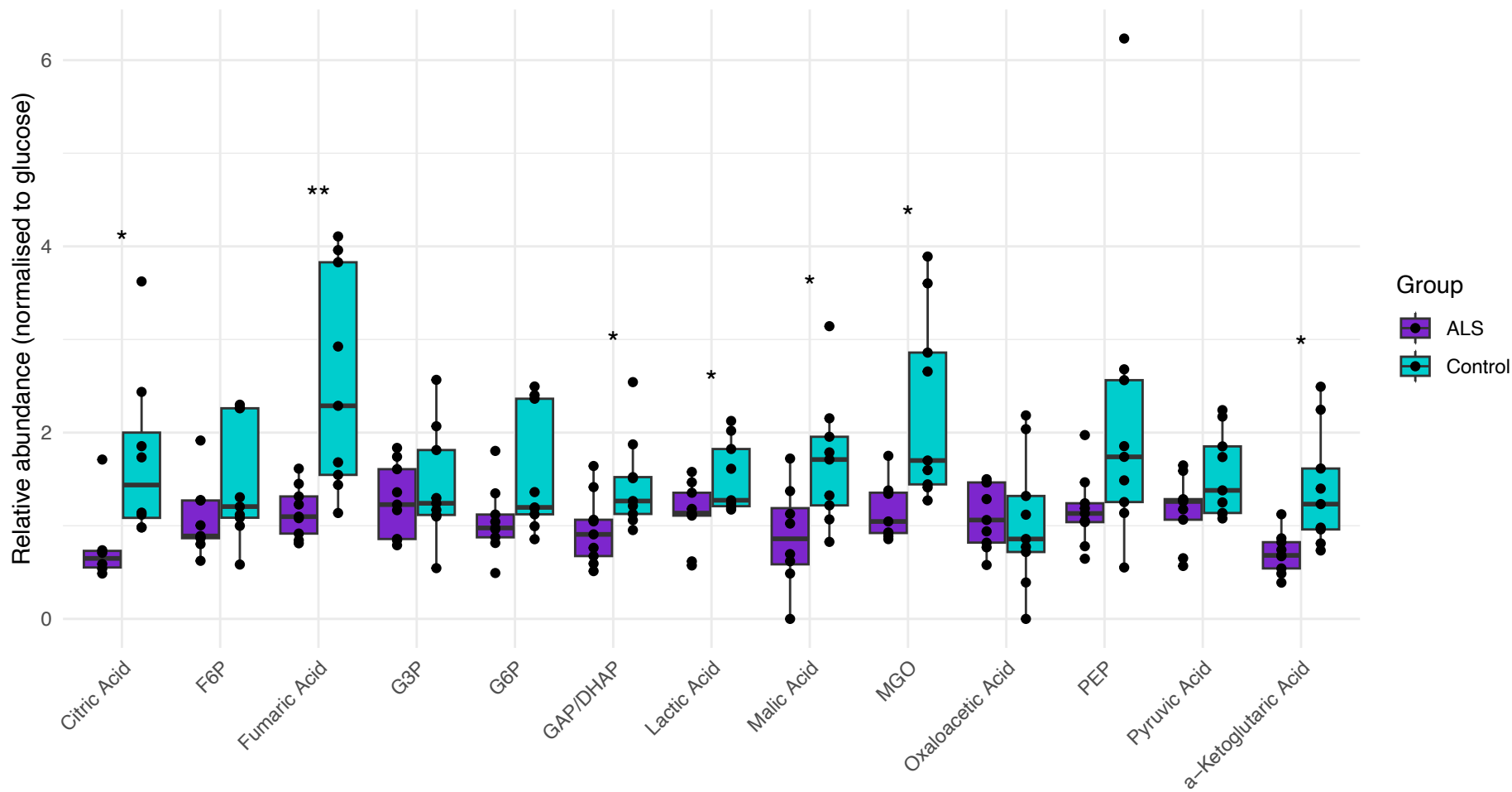

Supplement: Supplementary file 8 — Additional file 8: Supplementary Fig. 6. Fructose metabolism is deficient in C9orf72 + astrocytes. The effect of fructose supplementation on control and C9of72 + iAstrocytes metabolite levels by LC–MS analysis. Three control and three C9orf72 lines were analysed in triplicate. Significance determined via Welch’s t-test. *P < 0.05; **P < 0.01. [file 12916_2026_4727_MOESM8_ESM.pdf]
